# Predictors of individual mental health and psychological resilience after Australia’s 2019–2020 bushfires

**DOI:** 10.1177/00048674231175618

**Published:** 2023-06-01

**Authors:** Emily Macleod, Timothy Heffernan, Lisa-Marie Greenwood, Iain Walker, Jo Lane, Samantha K Stanley, Olivia Evans, Alison L Calear, Tegan Cruwys, Bruce K Christensen, Tim Kurz, Emily Lancsar, Julia Reynolds, Rachael Rodney Harris, Stewart Sutherland

**Affiliations:** 1 School of Medicine and Psychology, The Australian National University, Canberra, ACT, Australia; 2 Centre for Mental Health Research, The Australian National University, Canberra, ACT, Australia; 3 School of Built Environment, University of New South Wales, Sydney, NSW, Australia; 4 Melbourne Centre for Behaviour Change, The University of Melbourne, Parkville, VIC, Australia; 5 National Centre for Epidemiology and Population Health, The Australian National University, Canberra, ACT, Australia; 6 School of Psychological Science, The University of Western Australia, Perth, WA, Australia; 7 Centre for Entrepreneurial Agri-Technology, The Australian National University, Canberra, ACT, Australia

**Keywords:** Mental health, psychological resilience, wellbeing, bushfire, natural disaster

## Abstract

**Aims:**

We assessed the mental health effects of Australia’s 2019–2020 bushfires 12–18 months later, predicting psychological distress and positive psychological outcomes from bushfire exposure and a range of demographic variables, and seeking insights to enhance disaster preparedness and resilience planning for different profiles of people.

**Methods:**

We surveyed 3083 bushfire-affected and non-affected Australian residents about their experiences of bushfire, COVID-19, psychological distress (depression, anxiety, stress, post-traumatic stress disorder) and positive psychological outcomes (resilient coping, wellbeing).

**Results:**

We found high rates of distress across all participants, exacerbated by severity of bushfire exposure. For people who were bushfire-affected, being older, having less financial stress, and having no or fewer pre-existing mental disorders predicted both lower distress and higher positive outcomes. Being male or having less income loss also predicted positive outcomes. Severity of exposure, higher education and higher COVID-19-related stressors predicted both higher distress and higher positive outcomes. Pre-existing physical health diagnosis and previous bushfire experience did not significantly predict distress or positive outcomes.

**Recommendations:**

To promote disaster resilience, we recommend investment in mental health, particularly for younger adults and for those in rural and remote areas. We also recommend investment in mechanisms to protect against financial distress and the development of a broader definition of bushfire-related impacts than is currently used to capture brushfires’ far-reaching effects.

## Introduction

Between August 2019 and March 2020, Australia experienced one of the worst bushfire seasons in its history. The fires killed 33 people; burned 24 million hectares of land; and massively damaged flora, fauna, ecosystems, homes and businesses (Royal Commission into National Natural Disaster Arrangements, 2020). Climate change will make bushfires more frequent, more extreme and interspersed with other disasters (Intergovernmental Panel on Climate Change [IPCC], 2022). Disasters have significant and long-term impacts on mental health and wellbeing (Newnham et al., 2022); reducing these impacts is a priority in (Australia’s Department of Home Affairs, 2018). It is important, therefore, to understand how the 2019–2020 bushfires affected mental health to enable more effective delivery of support and to improve disaster resilience.

Following disasters such as bushfires, about one-third of adults experience significant psychological distress, which can continue for months or years (Bonanno et al., 2010). Distress after disaster is not inevitable though; most people recover or show resilience (Bonanno, 2005; Fletcher and Sarkar, 2013; Norris et al., 2009). Resilience refers to maintaining, adapting or returning quickly to adequate daily functioning after an event such as environmental disaster (Bonanno, 2005). The concept is usually captured by measuring the absence of psychological distress such as symptoms of depression, anxiety or post-traumatic stress disorder (PTSD) (Bonanno, 2004; Bryant et al., 2021; Lowe et al., 2015) and/or by measuring the presence of positive psychological characteristics, including coping with stress adaptively (Bonanno et al., 2011; Li et al., 2012).

Fully appreciating the impacts of disasters requires *concurrently* assessing *both* distress and positive psychological outcomes (e.g. resilient coping, wellbeing). It is feasible, for example, that someone who experienced PTSD after a traumatic event may simultaneously demonstrate wellbeing in other domains (Southwick et al., 2014); such strengths could be harnessed to improve wellbeing. Conversely, people who do not demonstrate clinical levels of psychological distress could have low wellbeing, which predicts poorer long-term health and wellbeing outcomes (Martín-María et al., 2017).

There is scant research regarding psychological resilience after bushfire. Previous Australian research found higher bushfire exposure and stress were the main predictors of clinical levels of psychological distress 3–4 years after the 2009 Victorian Black Friday bushfire and remained important predictors 10 years later (Bryant et al., 2014, 2021). Age and higher PTSD symptoms predicted fewer positive resilience characteristics 5 years after the Canadian Fort McMurray Wildfire (Adu et al., 2022). In the year following the 2019–2020 Australian bushfires, high levels of psychological distress were recorded (Usher et al., 2022), but predictors have not yet been identified.

Most of our knowledge about predictors of resilience after disaster comes from Northern Hemispheric research on non-bushfire disasters (e.g. hurricanes and earthquakes; for reviews, see Bonanno et al., 2010; Chen et al., 2020). These studies have often relied on small samples and lacked a comparison group. Factors predicting the absence of psychopathology or the presence of positive psychological outcomes include *demographic characteristics* such as being male, older, having higher education and higher income (Ikizer et al., 2016; Li et al., 2012; Ni et al., 2015; Silveira et al., 2021); the absence of pre-existing *financial* or *clinical stressors* (mental or physical health) (Adu et al., 2022; Ikizer et al., 2016; Ni et al., 2015); and experiencing lower disaster severity (Chan and Rhodes, 2014). Research has typically not assessed these predictors together to identify how they relate to both psychological distress and positive psychological outcomes, nor in the context of bushfires. The IPCC’s warning of increased disasters due to climate change makes developing a mental health and disaster research-base critical, especially in under-studied countries such as Australia.

Our study surveyed a large nationwide sample 12–18 months after the 2019–2020 bushfires. To assess mental health effects, we included both fire-affected and non-affected samples. For those affected by bushfire, we also aimed to evaluate individual demographic (age, gender, education, income), clinical (prior mental and physical health diagnoses), financial (stressors) and bushfire-related predictors of two facets of resilience: (1) psychological distress symptoms (depression, anxiety, PTSD, stress) and (2) the presence of positive psychological outcomes (resilient coping, wellbeing). We measured distress and positive outcomes simultaneously, expecting that predictors may differ for positive and negative facets of resilience. Understanding the specific factors that contribute to bushfire risk and resilience will allow for recommendations regarding ongoing support and future bushfire planning.

## Materials and methods

### Participants

Australian adults from both bushfire- and non-bushfire-affected regions completed survey questions about their individual demographic characteristics, mental health, wellbeing, and bushfire experiences, 12–18 months after the 2019–2020 bushfires. Participants were recruited through several methods to mitigate selection biases: a paid Qualtrics panel sample, purposive and convenience sampling (social media, word-of-mouth, emergency response and recovery organisations, local media stories, university study pools), and postal invitations. Participants completed the survey online or on paper. Participants provided informed consent by reading a participant information sheet and submitting a completed survey. Ethics approval was granted by the Australian National University Human Ethics Committee (#2020/591). After removing participants who did not meet minimum completion requirements, gave contradictory or unreliable responses, or had completion times more than 2 standard deviations below the mean completion time, our final sample was 3083 participants (see Supplementary Materials for further details, p. 1).

Demographic characteristics included postcode, gender, age, income and education (8-point scale from 0 = *no education* to 7 = *master’s, PhD or equivalent*). We present the participant characteristics across bushfire exposure levels at the start of the ‘Results’ section.

#### Mental health and wellbeing

We selected measures of psychological distress and positive psychological outcomes that were psychometrically sound, common to the literature and easy to administer online.

*Depression* was measured using the nine-item Patient Health Questionnaire (PHQ9) (Kroenke et al., 2001), which identifies symptoms over the previous 2 weeks corresponding to the *Diagnostic and Statistical Manual of Mental Disorders*, 5th Edition (*DSM*-5) (American Psychiatric Association, 2013) criteria for Major Depressive Disorder. Response options ranged from 0 (*not at all*) to 3 (*nearly every day*), with total scale scores ranging from 0 to 27 (α = 0.98).

*Anxiety* was measured using the seven-item General Anxiety Disorder (GAD7), which uses the same response format as the PHQ9 and identifies symptoms corresponding to the *DSM*-5 criteria for Generalised Anxiety Disorder (Spitzer et al., 2006). Total scale scores ranged from 0 to 21 (α = 0.97).

*Stress* was measured by the four-item Perceived Stress Scale (PSS4), which identifies respondents’ appraisals of unpredictability, uncontrollability and sense of being overwhelmed within their life in the last month (Cohen et al., 1983). Items were rated on a scale from 0 (*never*) to 4 (*very often*), with scale scores ranging from 0 to 16 (α = 0.68) (Cohen et al., 1983).

*Post-traumatic Stress* was measured among participants who were exposed to fire or smoke (i.e. fitting the *DSM*-5 criterion for a category A traumatic event required to classify PTSD) by completing the eight-item Post-traumatic Stress Disorder Index (PTSDI8) (Hansen et al., 2010). This identifies how often people experience symptoms of intrusion, avoidance and hypervigilance corresponding to the *DSM*-5 criteria for PTSD on a scale from 1 (*not at all*) to 4 (*most of the time*), with total scores ranging from 4 to 32 (α = 0.93).

*Psychological wellbeing* was measured by the World Health Organisation’s five-item Wellbeing Index (WHO5) (Topp et al., 2015). The five items ask about positively framed symptoms of wellbeing in the past 2 weeks, using a scale from 5 (*all of the time*) to 0 (*none of the time*). The sum is multiplied by 4 to give a final score from 0 to 100 (α = 0.89). Higher scores indicate greater wellbeing and lower mortality risk (Birket-Smith et al., 2009).

*Resilient coping* was measured by the four-item Brief Resilient Coping Scale (BRCS4) (Sinclair and Wallston, 2004), which identifies tendencies to adaptively cope with stress by asking questions about current coping behaviours on a scale from 1 (*does not describe me at all*) to 5 (*describes me very well*). Scores ranged from 4 to 20 (α = 0.70).

#### Other stressors

*Prior bushfire exposure* was measured using six items developed for the survey, asking participants to indicate (yes/no), whether they had prior to August 2019 experienced: a fire nearby, bushfire evacuation, loss or damage to property, threatened safety, fought fires or had other direct contact with fire (e.g. protecting property). Total prior bushfire exposure was calculated by summing scores (range 0–6).

*Prior mental or physical health diagnoses* were measured by asking participants to indicate (yes/no) if, prior to the 2019–2020 bushfires, they had ever received a *mental health diagnosis* for each of anxiety, depression, obsessive-compulsive disorder, schizophrenia, alcohol use, substance use disorder or PTSD (summed, total range: 0–7), and/or a *physical health diagnosis* for each of asthma, gastrointestinal disorder, chronic obstructive pulmonary disease or another health condition (summed, total range: 0–4).

*Reduced income* was measured by asking, ‘How has your household income been affected as a result of’ (1) bushfire and (2) COVID-19 (coronavirus disease). Participants responded on a scale from 1 (*much less income*) to 5 (*a lot more income*). Responses were dichotomised into two variables representing reduced income versus no reduced income for each of bushfire and COVID-19.

*COVID-19 Impact* was measured using the five-item Work and Social Adjustment Scale (WSAS) (Mundt et al., 2002), a validated scale measuring the impact of a problem (specified here as the COVID-19 pandemic) on the ability to carry out daily life activities. Responses were on a scale from 1 (*not impaired at all*) to 8 (*very seriously impaired*). Total scale scores ranged from 5 to 40 (α = 0.89).

#### Bushfire exposure

Participants answered 18 questions developed for the survey regarding experiences relating to bushfire, including whether they had experienced (yes/no) direct threat, disruption or loss due to bushfire; had been involved in bushfire response activities or services; or had indirect exposure to bushfire (see Supplementary Materials, p. 2, for specific items). The items were drawn from literature regarding measurement of disaster exposure (Bryant et al., 2014; Chan and Rhodes, 2014; Elal and Slade, 2005) and tailored to capture the unique aspects relevant to bushfire. Total Bushfire Exposure was calculated by summing all endorsed bushfire exposure experiences (0–18).

Respondents’ bushfire exposure was then categorised by severity: for those affected, severity was calculated to be low, medium or high (adapted from prior bushfire research; Bryant et al., 2014). For those not affected, indirect or non-affected categories were applied. *Low* severity exposure applied if participants were in an area with high bushfire alert levels, lost one or more community buildings (e.g. workplace), or were involved in fighting fires or providing a service in response to the fires as a professional (i.e. not personal impact) (*n* = 507). *Medium* severity exposure applied to those who experienced evacuation; lost personal property, pets or farm animals; moved to a new home; lost income while living in a bushfire-affected region; or if their partner or child experienced a major injury (*n* = 666). *High* severity exposure applied to those who experienced major injury, one or more deaths of a person close to them, felt their life was in danger, lost their home, or remained displaced since the fire (*n* = 424). Participants were categorised as having *Indirect* exposure if they were not in a bushfire-affected postcode or involved in the bushfire, but reported loss of income related to bushfire (*n* = 435). Participants were categorised as *Non-affected* (i.e. controls) if they did not live in a bushfire-affected postcode and did not endorse any of our 18 items assessing bushfire exposure (*n* = 1044). Participants were categorised into the highest category in which they endorsed one or more items.

## Results

### Participant characteristics

In total, 52% of participants reported low to high bushfire exposure, and a further 14% were indirectly affected by bushfire due to loss of income (see Table 1). Participants’ geographic representations aligned with areas of bushfire impact, with more bushfire-exposed participants from New South Wales and the Australian Capital Territory, and more non-exposed participants from Queensland and Western Australia, and representation from the remaining states in line with census data, χ^2^(9) = 250.3, *p* < 0.001, φ = 0.29. Proportionately more bushfire-exposed participants were from regional, rural and remote areas, and more non-exposed participants were from cities, χ^2^(4) = 83.7, *p* < 0.001, φ = 0.18. Compared to census data, our sample included an over-representation of Indigenous participants, who are more likely to reside in areas impacted by bushfire (Williamson, 2022). Indigenous participants were more likely to be bushfire-exposed (82%), χ^2^(1) = 129.1, *p* < 0.001, φ = 0.34.

**Table 1.** Demographic characteristics of bushfire-affected and non-affected participants, with comparisons to Australian census data, where available.

Characteristic	Total *N* *=* 3083		Bushfire-affected (high, medium, low) *n* = 1597 (52%)		Non-affected (indirect, control) *n* *=* 1479 (48%)		Census data 2021
*n* (valid total)	*n*	%	*n*	%	*n*	%	%
Bushfire exposure							
Category *n* = 3076							
High	424	13.8					
Medium	666	21.7					
Low	507	16.5					
Indirect	435	14.1					
Non-exposed	1044	33.9					
State *N* = 3083							
Australian Capital Territory	237	7.7	130	8.1	103	7	1.8
New South Wales	933	30.3	642	40.2	291	19.7	31.8
Victoria	728	23.6	378	23.7	350	23.7	25.6
Queensland	477	15.5	175	11	302	20.4	20.3
Tasmania	102	3.3	44	2.8	58	3.9	2.2
Western Australia	379	12.3	101	6.3	276	18.7	10.5
Northern Territory	14	0.5	6	0.4	8	9.5	0.9
South Australia	212	6.7	120	7.5	91	6.2	7.0
Gender *n* = 3082							
Male	1027	33.3	608	38.1	417	28.2	49.3
Female	2024	65.7	974	61	1047	70.8	50.7
Other	22	0.6	11	0.7	9	0.6	–
Prefer not to say	9	0.3	3	0.2	6	0.4	
Age *n* = 3083							
18–24	841	24.6	367	23	470	31.81	est 7.3
25–34	569	16.6	301	18.8	267	8.1	14.3
35–44	642	18.8	396	24.8	245	16.6	13.7
45–54	347	10.1	207	13.0	140	9.5	12.7
55–64	340	9.9	182	11.4	158	10.710.2	11.9
65–74	267	7.8	116	7.3	151	2.8	9.7
75–84	67	2	25	1.6	42	0.4	5.4
85+	10	0.3	3	0.2	6	31.81	2.1
Ethnicity *n* = 2879							
European/Caucasian	1956	57.2	1031	64.4	925	65.6	81
African and Middle Eastern	146	3.3	99	3.2	47	4	4.4
Asian	274	11.3	121	12	153	13.3	17.4
Peoples of the Americas	60	1.7	44	2.8	16	1.1	1.4
Māori and Pacific Islanders	98	2.8	60	3	38	2.1	2
Other	18	0.5	8	0.5	10	0.7	
Indigenous							
Aboriginal	237	6.9	183	11.5	54	4.5	2.9
Torres Strait Islander	60	1.8	58	3.6	2	0.2	0.1
Aboriginal and Torres Strait Islander	29	0.8	25	1.6	4	0.3	–
SES (IRSAD decile) *n* = 2744							
1	237	8.6	128	9	109	8.2	10
2	326	11.9	187	13.2	139	10.5	10
3	222	8.1	147	10.3	75	5.7	10
4	264	9.6	149	10.5	115	8.7	10
5	207	7.5	117	8.2	90	6.8	10
6	249	9.1	113	7.9	136	10.3	10
7	211	7.7	98	6.9	113	8.5	10
8	360	13.1	181	12.7	179	13	10
9	365	13.3	162	11.4	203	15.4	10
10	303	11	140	9.8	163	12.3	10
Remoteness *n* = 2744							
Major city	1608	58.6	726	51.1	882	66.7	72
Inner regional	614	22.4	354	22.2	260	19.7	18
Outer regional	425	15.5	286	17.9	139	9.4	8
Remote	66	2.4	43	2.7	23	1.7	1.1
Very remote	31	1.1	13	0.8	18	1.4	0.8
Annual household income (2018–2019) *n* = 2171							
Up to 25,999	414	19.1	214	17.8	200	20.6	15.4
$26,000–$41,599	202	9.3	100	8.3	102	10.5	9.8
$41,600–$64,999	380	17.5	182	15.1	198	20.4	12.8
$65,000–$90,999	431	19.9	279	23.2	152	15.7	13.9
$91,000–$155,999	500	23	303	25.2	197	20.3	23.8
$156,000+	244	11.2	124	10.3	120	12.4	22.6
Relationship status *n* = 2521							
Single	805	31.9	357	27.1	448	37.2	–
Partnered	482	19.1	263	20	219	18.2	–
De facto	194	7.7	89 6	8	105	8.7	11.5
Married	892	35.4	524	39.8	368	30.6	46.5
Separated	61	2.4	35	2.7	26	2.2	3.2
Widowed	61	2.4	38	2.9	23	1.9	5
Other	26	1	11	0.8	15	1.2	–
Education *n* = 2500							
Up to high school	791	31.6	351	26.9	440	26.9	37.6
Trade, apprenticeship, skills training, certificate or diploma	496	19.8	253	19.4	243	20.3	25.5
Some university	311	12.4	179	13.7	132	11	–
University graduate	882	35.3	512	39.3	370	30.9	26.3
Other	20	0.8	9	0.7	11	0.9	–
Fought fires *n* = 478							
Male			263	16.5			
Female			215	13.5			
Professional firefighters *n* = 244							
Male			148	9.3			
Female			96	6.0			

Sample sizes change across variables and bushfire-affected categories due to missing data. % = percent of total valid sample within each category column. Census data based on the Australian Bureau of Statistics 2021 census, retrieved from abs.gov.au/census (the 2016 census was used for Ethnicity data due to lack of 2021 data). Community demographics were derived from postcode, based on data from the Australian Bureau of Statistics (2016) Census. SES was derived via the SEIFA IRSAD (range: 1–10, with higher scores representing more advantage) and level of remoteness via the Australian Statistical Geography Standard Australian Statistical (ASGS) remoteness structure (range: 1–4, with higher scores reflecting greater urbanness).

SES: socioeconomic status; IRSAD: Index of Relative Socio-economic Advantage and Disadvantage.

Bushfire-exposed participants were older, *t*(2932) = 0.86, *p* < 0.001, *d* = 0.03, and had a lower average socioeconomic status (SES) (Index of Relative Socio-economic Advantage and Disadvantage [IRSAD]), *t*(2742) = 5.2, *p* < 0.001, *d* = 0.20, than non-exposed participants. Both groups had an over-representation of non-males, but less so in the non-exposed group, χ^2^(1) = 33.9, *p* < 0.001, φ = −0.11 (see Supplementary Materials, p. 4, for treatment of gender); 43% of bushfire-affected male participants fought fires, compared to 22% of bushfire-affected female participants. Bushfire-affected participants were more educated than non-affected participants, *t*(2498) = 5.4, *p* < 0.001, *d* = 0.22.

In contrast to the high rate of self-reported bushfire experiences (52%; *n* = 1597), only 18.5% of participants met criteria for government bushfire-related assistance based on government classification of bushfire-affected postcodes (*n* = 569). Table 2 shows the significant lower likelihood of participants being classified by government as exposed, χ^2^(2) = 354.33, *p* < 0.001. See Supplementary Materials (p. 5) for further analyses by exposure category.

**Table 2.** Participants categorised as fire-affected and non-affected based on postcode compared to self-reported experiences.

Self-report measure of exposure	Postcode-based measure of exposure		Total	%
	Non-affected	Fire-affected		
Non-affected	970_a_	74_b_	1044	33.9
Indirectly affected	435_a_	0_b_	435	14.1
Fire-affected (high, medium or low)	1102_a_	495_b_	1597	51.9
Total	2507	569	3076	
%	81.5	18.5		

Each subscript letter denotes a subset of postcode-based exposure categories whose column proportions do not differ significantly from each other at the 0.05 level.

### Psychological distress and positive psychological outcomes

**Figure 1.**
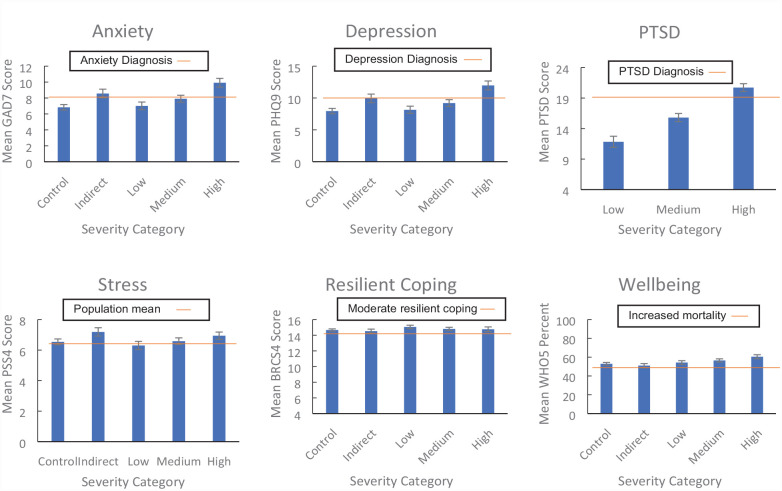
Distress and positive psychological characteristics as a function of bushfire exposure. Reference lines are based on empirically derived cutoff scores for probable clinical diagnoses relating to Depression (Kroenke et al., 2001), Anxiety (Plummer et al., 2016) and PTSD (Hansen et al., 2010); recommended categories for resilient coping (Sinclair and Wallston, 2004); population means for stress (Warttig et al., 2013); and the wellbeing cutoff (below the line) for increased likelihood of higher mortality (Birket-Smith et al., 2009). See Supplementary Materials (p. 11) for further details about specified cutoffs and significant group differences.

We assessed each outcome measure across severity of bushfire exposure using a series of one-way analyses of variance (ANOVA) with Tukey’s and Games–Howell post hoc tests of group differences (see Table 3). Results are reported here if *p* < 0.05 (full analyses in Supplementary Materials, pp. 7–11, including missing data treatment, p. 6). There were significant main effects of severity of bushfire exposure on all measures of depression, anxiety, stress, PTSD, wellbeing and resilient coping (η^2^ range = 0.004–0.25). As shown in Figure 1, participants with High exposure had significantly higher depression and anxiety scores compared to all other severity groups, and high levels of stress (significantly higher than the Low group), *p* < 0.05 to *p* < 0.001. Wellbeing scores were also highest in the High group compared to all other groups, followed by participants in the Medium group having higher wellbeing scores than Indirect or Control participants, *p* < 0.05 to *p* < 0.001. Participants in Medium and Indirect groups had the next highest depression and anxiety scores, significantly higher than Control participants, *p* < 0.001. The Indirect group had the highest levels of stress, significantly higher than the levels of the Medium, Low and Control participants, *p* < 0.05 to *p* < 0.001.

**Table 3.** Means (SD) and correlations for measures of psychological distress and positive psychological outcomes for total participant sample.

Variable (survey measure)	*n*	M	SD	Range	α	1	2	3	4	5
1. Depression (PHQ9)	3066	9.09	6.95	0–27	0.98	–				
2. Anxiety (GAD7)	3070	7.77	5.67	0–21	0.97	0.855**	–			
3. Stress (PSS4)	3074	6.66	2.92	0–16	0.68	0.625**	0.654**	–		
4. Resilient coping (BRCS4)	3079	14.75	2.60	4–20	0.70	−0.181**	−0.184**	−0.333**	–	
5. Wellbeing (WHO5)	3072	54.71	22.33	0–100	0.89	−0.447**	−0.425**	−0.589**	0.370**	–
6. PTSD (PTSDI8)	830	16.68	6.66	4–32	0.93	−0.472**	−0.466**	0.220**	0.015	0.066

SD: standard deviation; PHQ9: nine-item Patient Health Questionnaire; GAD7: seven-item General Anxiety Disorder; PSS4: four-item Perceived Stress Scale; BRCS4: four-item Brief Resilient Coping Scale; WHO5: World Health Organisation’s five-item Wellbeing Index; PTSD: post-traumatic stress disorder; PTSDI8: eight-item Post-traumatic Stress Disorder Index. **p < 0.001.

Among those exposed to bushfire, PTSD scores differed significantly between each severity group (*p* < 0.001), with participants in the High exposure group showing the highest mean PTSD scores, followed by participants in the Medium group, then the Low group.

Across all participants, males reported significantly higher scores on resilient coping (Cohen’s *d* = 0.25) and wellbeing (*d* = 0.53), and lower scores on stress (*d* = −0.33), anxiety (*d* = −0.26) and depression (*d* = −0.22) than non-males (see Supplementary Materials for analyses, p. 11). However, males had higher PTSD scores than non-males (*d* = 0.21)

Compared to non-Indigenous Australians, Indigenous participants had higher scores of depression, anxiety and PTSD; similar scores on stress; and *higher* scores on resilient coping and wellbeing (see Table 4).

**Table 4.** Comparison of Means (SD) for measures of psychological distress and positive psychological outcomes for non-Indigenous and Indigenous participants.

Variable (survey measure)	Non-Indigenous			Indigenous			*t*	*df*	*d*
	*n*	M	SD	*n*	M	SD			
Depression (PHQ9)^a^	2196	8.56	6.82	325	11.23	7.38	−6.16**	410.26	0.39
Anxiety (GAD7)^a^	2197	7.39	5.57	325	9.00	5.95	−4.59**	412.55	−0.29
Stress (PSS4)	2198	6.61	2.93	326	6.42	3.03	1.13	2522	−0.05
Resilient coping (BRCS4)^a^	2199	14.70	2.49	326	15.57	2.90	−5.18**	399.25	−0.34
Wellbeing (WHO5)	2199	53.67	21.76	324	67.53	23.33	−10.60**	2521	−0.63
PTSD (PTSDI8)^a^	611	15.68	6.4	142	21.19	5.76	−10.05**	229.11	−0.88

SD: standard deviation; PHQ9: nine-item Patient Health Questionnaire; GAD7: seven-item General Anxiety Disorder; PSS4: four-item Perceived Stress Scale; BRCS4: four-item Brief Resilient Coping Scale; WHO5: World Health Organisation’s five-item Wellbeing Index; PTSD: post-traumatic stress disorder; PTSDI8: eight-item Post-traumatic Stress Disorder Index.

a Based on Levene’s test, equal variances were not assumed, so Welch’s *t*-test and Hedge’s correction for effect size were used.

** *p* < 0.001.

### Predictors of psychological distress and resilience among those affected by bushfires

Given the correlations among our outcome measures (Table 3), we used factor analysis to reduce measures to two factors: Positive Psychological Outcomes (Positive) and Psychological Distress (Distress) (see Supplementary Materials, pp. 15–19, for justification and full analyses). Factor scores were used in all subsequent analyses. The two factors were weakly associated with each other (*r* = 0.113, *p* = 0.001).

Age, gender, education, income, financial stress, COVID-19 stressors, pre-existing physical health diagnosis, pre-existing mental health diagnosis, prior bushfire exposure and 2019–2020 bushfire exposure were all significantly correlated with either the Distress or Positive factor scores, or both (*r*s range from 0.07 to 0.47, all *p*s < 0.01; see Supplementary Materials, p. 20).

We used a hierarchical multiple linear regression to determine if, after controlling for individual demographic variables (age, education, gender, income), COVID-19 stressors, and clinical and financial stressors (Step 1), severity of bushfire exposure (Step 2) contributed to the prediction of Distress and Positive outcome scores. There were 712 bushfire-affected participants who had valid data for all of the outcome measures and predictors. See Tables 5 and 6 for results for each regression model.

**Table 5.** Hierarchical linear regression analysis results for variables predicting psychological distress after bushfire.

Variable	Model 1			Model 2		
	*B*	*SE B*	β	*B*	*SE B*	β
Age	−0.02	0.00	−0.33**	−0.02	0.00	−0.282**
Gender	−0.08	0.06	−0.04	0.03	0.06	0.01
Education	0.06	0.02	0.11**	0.04	0.02	0.07*
Income loss	−0.02	0.01	−0.06	−0.01	0.01	−0.05
Covid stressor	0.01	0.00	0.06	0.00	0.00	0.02
Financial threat	0.06	0.01	0.30**	0.06	0.00	0.30**
Prior mental health diagnosis	0.15	0.03	0.22**	0.16	0.02	0.22**
Prior physical health diagnosis	0.05	0.04	0.04	−0.02	0.04	−0.02
Prior fire exposure				−0.00	0.01	−0.01
2019–2020 fire exposure severity				0.09	0.01	0.27**

*R*^2^ = 0.41 for Step 1; ∆*R*^2^ = 0.06 for Step 2.

* *p* < 0.05. **p < 0.001.

**Table 6.** Hierarchical linear regression analysis results for variables predicting positive psychological outcomes after bushfire.

Variable	Model 1			Model 2		
	*B*	*SE B*	β	*B*	*SE B*	β
Age	0.00	0.00	0.04	0.00	0.00	0.08*
Gender	−0.51	0.07	−0.25**	−0.42	0.07	−0.21**
Education	0.11	0.02	0.21**	0.09	0.02	0.17**
Income loss	0.02	0.01	0.08*	0.02	0.01	0.08*
Covid stressor	0.02	0.00	0.17**	0.02	0.00	0.14**
Financial threat	−0.02	0.01	−0.11*	−0.02	0.01	−0.11*
Prior mental health diagnosis	−0.09	0.03	−0.13*	−0.08	0.03	−0.12*
Prior physical health diagnosis	0.02	0.05	0.01	−0.04	0.05	−0.03
Prior fire exposure				−0.01	0.02	−0.01
2019–2020 fire exposure severity				0.07	0.01	0.23**

*R*^2^ = 0.19 for Step 1; ∆*R*^2^ = 0.05 for Step 2.

* *p* < 0.05. **p < 0.001.

The full model strongly predicted Distress symptoms, adjusted *R*^2^ = 0.46, *F*(10, 701) = 62.44, *p* < 0.001, including a statistically significant amount added on Step 2 by total bushfire exposure and previous fire exposure, adjusted *R*^2^ of 0.06, *F*(2, 701) = 38.73, *p* < 0.001. In the final model, the significant predictors of Distress were younger age, higher education, more financial stress, more pre-existing mental health diagnoses and higher total bushfire exposure. Gender, income loss, COVID-19 stressors, pre-existing physical health diagnosis and previous fire exposure were not significant predictors of Distress.

The full model also predicted Positive outcomes, *R*^2^ = 0.23, *F*(10, 701) = 22.70, *p* < 0.001, including a statistically significant amount added on Step 2 by total bushfire exposure and previous fire exposure, adjusted *R*^2^ of 0.05, *F*(2, 701) = 21.85, *p* < 0.001. The significant predictors of Positive outcomes were older age, male gender, higher education, higher less income loss lower financial stressors, fewer pre-existing mental health diagnoses, higher COVID-19 stressors and higher exposure severity. Pre-existing physical health diagnoses and previous fire exposure were not significant predictors of Positive scores.

## Discussion

Bushfire-affected and non-affected participants alike reported significant distress: half met clinical cutoff criteria for Depression and/or Anxiety (Plummer et al., 2016), and 35% of bushfire-affected participants met the PTSD cutoff (Hansen et al., 2010). Greater bushfire exposure increased the likelihood of meeting any clinical cutoff. Across all bushfire-affected participants, being older, having less financial stress, or having no or fewer pre-existing mental health diagnoses predicted both lower distress and higher positive outcomes, consistent with international research (Bonanno et al., 2007; Chen et al., 2020). Being male or having less income loss also predicted positive outcomes. Fire-related experiences and accompanying distress were more widespread than was captured by the government’s postcode definition of affected areas.

Bushfire-affected respondents were more likely to be in regional or rural areas, reflecting the regions vulnerable to bushfire. Our survey included a high proportion of Aboriginal and Torres Strait Islander (Indigenous) peoples, who are over-represented in bushfire-affected regions. Bushfire and land damage threaten connection to land and culture (Williamson, 2022). Ongoing structural and social inequities position Indigenous peoples in Australia to be more vulnerable to the health and financial impacts of environmental disaster. Our survey showed that bushfire not only exacerbated existing mental health inequities for Indigenous peoples but also highlighted their resilience. These results reinforce the importance of moving beyond deficit approaches (Williamson, 2022). The disproportionate impact of bushfire on Indigenous Australians and the unique value of Indigenous knowledge, culture and practice in bushfire preparedness and response militate for greater Indigenous involvement in all aspects of bushfire management, including health promotion and service delivery.

### Theoretical implications

Similar sets of predictors related to both psychological distress and positive outcomes, indicating that experiences related to depression, anxiety, PTSD, wellbeing and resilient coping fit under a general construct of resilience. Psychological outcomes loaded onto two weakly related factors: positive psychological experiences and experiences of psychological distress. Although the two factors shared common predictors, the overlap was imperfect, implying that psychological distress and positive psychological outcomes are different constructs under the umbrella of resilience rather than different ends of a single distress continuum (consistent with Margraf et al., 2020).

Our research highlights the need to conceptualise and measure both distress and resilience comprehensively to understand how they can be harnessed to promote disaster resilience (Fletcher and Sarkar, 2013). Our model predicting positive outcomes explained less variance than that predicting distress, but also included more significant predictors, suggesting positive psychological experiences after disaster may be more diverse and contextual than experiences of distress. For example, severity of bushfire exposure predicted both more distress and more positive outcomes, and higher COVID-19 stressors predicted more positive outcomes, but not distress. These nuanced findings likely reflect the complex contextual nature of disaster experiences and coping responses. For example, disaster-affected individuals and communities often experience connection and growth due to shared trauma (Muldoon et al., 2019), and social connection supported mental health 12–18 months after bushfire in the current sample (Cruwys et al., under review).

### Implications and recommendations

Our results highlight the characteristics of those vulnerable to mental health impacts of bushfire in Australia and factors relating to psychological strength, suggesting priority areas to target for promoting disaster resilience. One key action likely to promote disaster resilience is mental health investment across each stage of the disaster management cycle (e.g. disaster mitigation, planning, response and recovery), especially supporting vulnerable groups, including those with existing mental health conditions, younger people and Indigenous peoples. Another is to improve financial security for all those financially affected by a disaster, not just in areas directly disaster-affected.

Our results underscore the need to review methods for measuring bushfire-affectedness to improve access to financial support for more Australians. A higher proportion of our sample reported bushfire impacts than those captured by postcode, the criteria required to access bushfire-related financial support. As financial threat predicted overall resilience, we recommend broadening the definition of those affected by bushfire, to reduce the wide-reaching impacts of financial distress. Given bushfire-affected people are more likely to live outside metropolitan areas, often with lower SES, prudent investment to improve financial stability would promote bushfire resilience.

### Limitations

Despite varied recruitment methods, the convenience sample may be non-representative. Also, the COVID-19 pandemic started in Australia a month after the fires ended, adding to distress. Compared to urban areas, bushfire-affected regions experienced fewer COVID-19 restrictions, but aspects of bushfire recovery were disrupted, including logistics and resources for rebuilding, and mental health support access. Hence, bushfire-affected participants may have been more vulnerable to mental health impacts of COVID-19, as cumulative stressors increase risk of psychological distress (Lowe et al., 2019).

## Conclusion

In line with other disaster research (Bryant et al., 2021; Usher et al., 2022), we provide further evidence that experiencing bushfire increases risk of psychological distress. As disasters become more frequent and extreme, robust policy and practice to support disaster resilience is essential, particularly given the immense pressure that mental health systems are currently experiencing (American Psychological Association [APA], 2022). Disasters exacerbate existing inequities, warranting strong investment in preventive mental health and promotion of financial stability, particularly in bushfire-prone regions. We have also identified groups with greater psychological resources supporting resilience; drawing on strengths among vulnerable groups should be considered in resilience planning (e.g. Indigenous-controlled health organisations providing culturally safe and appropriate health provision). Future longitudinal research is needed to understand unique profiles of risk and resilience to best support resilience for future disaster.

## Supplemental Material

sj-docx-1-anp-10.1177_00048674231175618 – Supplemental material for Predictors of individual mental health and psychological resilience after Australia’s 2019–2020 bushfires Click here for additional data file. Supplemental material, sj-docx-1-anp-10.1177_00048674231175618 for Predictors of individual mental health and psychological resilience after Australia’s 2019–2020 bushfires by Emily Macleod, Timothy Heffernan, Lisa-Marie Greenwood, Iain Walker, Jo Lane, Samantha K Stanley, Olivia Evans, Alison L Calear, Tegan Cruwys, Bruce K Christensen, Tim Kurz, Emily Lancsar, Julia Reynolds, Rachael Rodney Harris and Stewart Sutherland in Australian & New Zealand Journal of Psychiatry
