# Supplementary material for: Predictors of individual mental health and psychological resilience after Australia’s 2019–2020 bushfires
Source: Aust N Z J Psychiatry. 2023 Jun 1;58(1):58–69. doi: 10.1177/00048674231175618 (PMC10756019; doi:10.1177/00048674231175618)
Supplement: sj-docx-1-anp-10.1177_00048674231175618 – Supplemental material for Predictors of individual mental health and psychological resilience after Australia’s 2019–2020 bushfires [file sj-docx-1-anp-10.1177_00048674231175618.docx]

**SUPPLEMENTARY ANALYSIS AND DATA FILE**

Supplementary analysis and data are presented under headings corresponding with the sections in the main text.

**2 Materials and Methods**

**2.1 Participants**

**Table 1.**

*Mean (SD) Selected Demographic Characteristics and Outcome Scores by Recruitment Method*

|  | **General** | | | **University pool A**  **(smoke-affected region)** | | | | **University pool B**  **(non-affected region)** | | | | | | **Paid panel** | | | | | **Postal** | | | |  |  |
| --- | --- | --- | --- | --- | --- | --- | --- | --- | --- | --- | --- | --- | --- | --- | --- | --- | --- | --- | --- | --- | --- | --- | --- | --- |
| Variable | *N* | *M* | *SD* | | *N* | *M* | *SD* | | *N* | *MeaM* | *SD* | *N* | | | *M* |  | *SD* | | *N* | | *M* | *SD* | | |
| Age | 781 | 50.61 | 15.32 | | 53 | 20.62 | 5.347 | | 197 | 21.28 | 6.23 | | 2036 | | 36.69 | | | 15.85 | 16 | 63.69 | | 16.68 | |  |
| ^1^IRSAD | 705 | 4.91 | 2.81 | | 52 | 5.37 | 3.48 | | 184 | 7.35 | 2.51 | | 1787 | | 5.95 | | | 2.95 | 16 | 5.25 | | 2.80 | |  |
| ^1^Remoteness | 705 | 0.96 | 0.96 | | 52 | .33 | 0.59 | | 184 | 0.30 | 0.70 | | 1787 | | .57 | | | .87 | 16 | 1.06 | | .93 | |  |
| Resilient-Coping (BRCS4) | 779 | 14.92 | 2.37 | | 53 | 14.66 | 2.53 | | 196 | 14.57 | 2.46 | | 2035 | | 14.71 | | | 2.70 | 16 | 13.94 | | 2.11 | |  |
| Stress (PSS4) | 776 | 6.10 | 2.81 | | 53 | 7.47 | 2.94 | | 196 | 6.92 | 2.79 | | 2033 | | 6.83 | | | 2.93 | 16 | 7.06 | | 3.77 | |  |
| Wellbeing (WHO5 %) | 776 | 54.49 | 20.65 | | 53 | 49.81 | 19.16 | | 196 | 51.63 | 17.80 | | 2031 | | 55.26 | | | 23.32 | 16 | 49.75 | | 27.08 | |  |
| Anxiety (GAD7) | 777 | 5.99 | 4.87 | | 53 | 8.51 | 5.18 | | 196 | 7.85 | 4.73 | | 2028 | | 8.44 | | | 5.89 | 16 | 5.25 | | 5.48 | |  |
| Depression (PHQ9) | 776 | 6.72 | 5.46 | | 53 | 9.04 | 5.82 | | 196 | 8.09 | 5.43 | | 2025 | | 10.12 | | | 7.38 | 16 | 6.44 | | 6.20 | |  |

^1^Derived from data from the Australian Bureau of Statistics 2016 Census, based on postcode (Australian Bureau of Statistics, 2016). Socioeconomic status was measured via the SEIFA Index of Relative Socio-Economic Advantage and Disadvantage, and level of remoteness via the Australian Statistical Geography Standard Australian Statistical (ASGS) remoteness structure.

Note: *General* population sample recruited through convenience and purposive sampling methods.

**Table 2.**

*Results of One-Way ANOVAs of Selected Demographic Characteristics and Outcome Scores by Method of Recruitment*

|  |  |  |  |  |
| --- | --- | --- | --- | --- |
| Variable | df | F | *p* | Eta-squared |
| Age | 4 | 223.73 | <.001 | .225 |
| SES (Index of Relative Socioeconomic Advantage and Disadvantage) | 4 | 31.51 | <.001 | .044 |
| Remoteness | 4 | 35.31 | <.001 | .049 |
| Resilient coping (BRCS4 Total) | 4 | 1.44 | .184 | .002 |
| Stress (PSS4 Total) | 4 | 10.43 | <.001 | .013 |
| Wellbeing (WHO5 %) | 4 | 2.09 | .078 | .003 |
| Anxiety (GAD7 Total) | 4 | 28.33 | <.001 | .036 |
| Depression (PHQ9 Total) | 4 | 36.84 | <.001 | .046 |

**2.1.3** **Bushfire Exposure**

## Table 3.

*Severity of Bushfire Experience: Cumulative Bushfire Experiences Scale Scores and Severity of Bushfire Exposure Category*

| Scale variables in each exposure category | Theme | Supporting Literature | Frequency of experience (N) |
| --- | --- | --- | --- |
| HIGH CATEGORY |  |  |  |
| One cared for person died | Threat and disruption | (Sumer et al., 2005)  (Verger et al., 2003) | 87 |
| More than one cared for person died | Threat and disruption |  | 35 |
| Received a major physical injury or illness | Threat/harm | (Norris and Uhl, 1993)  (David et al., 1996)  (Elal and Slade, 2005) | 79 |
| Felt life was in immediate danger | Threat | (David et al., 1996)  (Ikizer et al., 2016)  (Polusny et al., 2008) | 147 |
| Home lost/damaged | Loss & disruption | (David et al., 1996)  (Elal and Slade, 2005) | 199 |
| Currently still displaced | Disruption | (Elal and Slade, 2005; David et al., 1996) | 64 |
|  |  |  |  |
| MEDIUM CATEGORY |  |  |  |
| Evacuation | Threat & disruption | (Ginexi et al., 2000)  (Polusny et al., 2008) | 437 |
| A partner, child, or close person received a major injury | Threat | (Norris and Uhl, 1993)  (Bountress et al., 2020)  (David et al., 1996)  (Elal and Slade, 2005) | 207 |
| Lost personal property | Loss | (Norris and Uhl, 1993)  (Elal and Slade, 2005)  Weihs, Simmens, & Hoyt, 2000) | 189 |
| Loss of farm animals | Loss |  | 89 |
| Loss of pets | Loss | (Bountress et al., 2020)  (Verger et al., 2003) | 76 |
| Moved home | Disruption | (David et al., 1996) | 84 |
| Lost income* | Loss | (Norris and Uhl, 1993)  (Ginexi et al., 2000) | 555 |
| LOW CATEGORY |  |  |  |
| Endorse one of top two alert levels | Threat |  | 1199 |
| Lost one or more of community buildings (e.g., place of worship, workplace, child’s school or day-care, relative or friend’s home, other homes or buildings in suburb) | Threat & Disruption |  | 296 |
| Fire fight (self) | Threat |  | 478 |
| Service provision | Threat/Vicarious trauma |  | 698 |
| Fire response | Threat/Vicarious trauma |  | 412 |

## *For participants who did not endorse any other bushfire-related experiences on the scale, and who were not in a fire-affected area (derived from postcode), lost income was used to categorise participants as Indirectly Affected.

In contrast to prior research, we found no evidence of impacts from cumulative bushfire exposure OR stress-inoculation occurring from prior fire exposure (for a review see Chen 2020).

**3 Results**

**3.1 Participant Characteristics**

**Treatment of Gender**

Due to the small number of transgender and non-binary gender responses, and the lack of statistical power to analyse these groups separately, gender was included in analyses as a binary variable, combining all non-male groups to facilitate inclusion rather than exclusion in analyses (Cameron et al., 2019), based on the rationale that non-male people tend to experience poorer mental health and wellbeing (Jones et al., 2019). We acknowledge that this occludes the experiences of participants who do not identify as male or female.

**Table 4.**

*Participants Categorised as Fire-affected and Non-affected Based on Postcode Compared to Self-reported Experiences.*

|  |  | Postcode-based measure of exposure | |  |  |
| --- | --- | --- | --- | --- | --- |
|  |  | Non-affected | Fire-affected | Total | % |
| Self-report measure of exposure | Non-affected | 970 _a_ | 74_b_ | 1044 | 33.9% |
|  | Indirectly affected | 435 _a_ | 0_b_ | 435 | 14.1% |
|  | Low | 401 _a_ | 106 _a_ | 1597 | 51.9% |
|  | Medium | 383 | 283 |  |  |
|  | Low | 318 | 106 |  |  |
|  | Total | 2507 | 569 | 3076 |  |
|  | % | 81.5% | 18.5% |  |  |

(*Χ*^2^(4) = 457.05, *p* < .001).

*Note*. Each subscript letter denotes a subset of postcode-based exposure categories whose column proportions do not differ significantly from each other at the .05 level.

## Psychological distress and positive psychological outcomes

## Data Screening

Prior to analysis, unusable data were removed from the dataset (*n=*857). Data were removed if respondents did not meet the minimum survey completion requirements, defined as providing responses to the initial question blocks containing primary mental health outcomes and residential postcode (required to indicate bushfire exposure); these blocks represented roughly 20% of total survey questions and were required to filter participants for bushfire exposure questions. Data were also removed if the survey completion time was more than 2 standard deviations below the mean time ( a requirement also enforced by the paid panel provider), or if participants provided contradictory response items (e.g., provided an inner-city residential postcode but selected a farm as their main residence type). Of the eligible survey respondents (*n*=3,940), 3,083 had usable data after screening.

**Missing data**

After meeting minimum inclusion requirements, between 13 and 18 participants had missing data for each of the six primary outcomes (see breakdown in table below). Primary outcomes were predominantly measured at the beginning of the survey, likely accounting for the low rates of missing responses. Data was assumed to be Missing At Random and a mean imputation (pro rata) method was used to maximise inclusion of data (Gale and Hawley, 2001; McKnight, 2007), which is appropriate when missing data is less than 10% (Fox-Wasylyshyn and El-Masri, 2005; Shrive et al., 2006). Scale score decisions relating to missing data were managed by creating a dummy variable to pro-rate missing data based on respondents' available scores (Gayle and Hawley, 2001). Using a dummy variable meant there were two variables for each scale item and no original data was written over. The average of the available scores was calculated and then imputed into the newly created (dummy) variable and total scores were then calculated. If participants did not enter responses for any of the scale responses, and therefore no scores were provided to create imputed values with, their data for the scale was deleted listwise.

**Table 5.**

*Participants with Partial Data in Each of the Outcome Measures of Distress and Resilience*

| Scale |  | *N*  Partial data | %  Partial data |
| --- | --- | --- | --- |
| Depression (PHQ9) |  | 15 | 1% |
| Anxiety (GAD7) |  | 18 | 1% |
| Stress  (PSS4) |  | 16 | 0.8% |
| Resilience-coping (BRCS) |  | 13 | 0.6% |
| Wellbeing (WHO5) |  | 13 | 0.8% |
| PTSD  (PTSDI8) |  | 13 | 3.0% |

Total Bushfire Exposure was a count of individual’s bushfire experiences developed for the study. Based on preliminary analyses of missing data, Total Bushfire Exposure data was assumed to be Missing at Random. Given that a) missing data had an implication on the meaning of the score (higher scores imply higher exposure), and b) there was no existing data to guide the appropriateness of inferring the presence of one experience based on others, to maintain the integrity of the Total Bushfire Exposure scores, respondents’ total score data was excluded if participants answered less than 80% of questions (i.e., a maximum of 2 missing data allowed for scales with 7 to 10 scale questions). When applying this cut-off criteria, 47 participants were removed due to excess missing data.

***ANOVAs of psychological distress and positive psychological outcomes as a function of category of severity of bushfire exposure***

**ANOVA Assumption tests**

Inspection of boxplots for values of greater than 1.5 box lengths from the edge of the box indicated that there were minimal outliers for WHO5, GAD7 and PHQ9 data and mild outliers consistently spread across each severity group for BRCS4 and PSS4 scores. Given the similar pattern of outliers across severity groups, and the feasibility that these were genuine scores, outliers were not excluded from the analysis. Shapiro-Wilk tests showed that outcome data was not normally distributed in any severity group (High, Medium, Low, Indirect Income, Control: <.001). Visual inspection of boxplots indicated that distributions of outcome scores were similar for all severity groups.

**ANOVA comparative analyses**

Given that our outcome data was non-parametric, we ran a series of Kruskal-Wallis H tests to determine if there were differences in the mean ranks of each outcome’s scores between the five severity levels: High, Medium, Low, Indirect Income, and Control. Distributions for each of the outcome scores for Depression, Anxiety, Resilience, Wellbeing, and PTSD scores were similar for all severity groups, as assessed by visual inspection of a boxplot. Median scores were statistically different significantly between severity groups for Depression, Anxiety, Wellbeing, and PTSD, smallest H(4) = 22.12, *p* < .001, but not Resilience, H(4) = 5.70, *p*= .223. Non-parametric Kruskal-Wallis H tests yielded the same results as One-Way ANOVAs so we reported the ANOVA results to enable the use of means for ease of interpretation of data.

## Table 6.

*Results for Separate One-way ANOVAs of Psychological Distress and Positive Psychological Outcomes as a Function of Severity of Bushfire Exposure Group*

| Measure | High | | | | Medium | | | | Low | | | | Indirect | | | Control | | | | | F (df) | | η^2^ | |
| --- | --- | --- | --- | --- | --- | --- | --- | --- | --- | --- | --- | --- | --- | --- | --- | --- | --- | --- | --- | --- | --- | --- | --- | --- |
|  | *N* | *M* | *SD* | *N* | | *M* | *SD* | *N* | | *M* | *SD* | *N* | | *M* | *SD* | | *N* | *M* | *SD* |  | |  | |  |
| Anxiety  (GAD7) | 422 | 9.92 | 5.62 | 665 | | 7.91 | 5.65 | 507 | | 7.01 | 5.43 | 431 | | 8.56 | 5.65 | | 1042 | 6.83 | 5.53 | 27.70 (4, 3062)** | | .04 | |  |
| Depression (PHQ9) | 422 | 11.97 | 7.17 | 664 | | 9.20 | 6.90 | 507 | | 8.14 | 6.43 | 429 | | 9.94 | 6.97 | | 1042 | 7.95 | 6.75 | 30.24 (4, 3059)** | | .04 | |  |
| Resilient coping (BRCS) | 423 | 14.76 | 3.09 | 666 | | 14.8 | 2.68 | 507 | | 15.06 | 2.46 | 434 | | 14.52 | 2.68 | | 1044 | 14.67 | 2.34 | 2.99 (4, 3069)* | | .004 | |  |
| Stress  (PSS4) | 422 | 6.94 | 2.44 | 666 | | 6.58 | 2.80 | 507 | | 6.30 | 3.00 | 433 | | 7.19 | 2.86 | | 1043 | 6.54 | 3.10 | 7.08 (4, 3066)** | | .01 | |  |
| Wellbeing (WHO5) | 422 | 60.49 | 21.59 | 655 | | 56.52 | 21.72 | 506 | | 54.30 | 21.91 | 433 | | 51.03 | 22.60 | | 1044 | 52.97 | 22.59 | 12.95 (4, 3065)** | | .02 | |  |
| PTSD  (PTSDI8) | 314 | 20.72 | 5.69 | 323 | | 15.80 | 6.01 | 125 | | 11.83 | 5.08 |  | |  |  | |  |  |  | 123.29 (2, 759)** | | .25 | |  |

** *p* <.001

** *p* <.01

**Table 7.**

*Post-hoc Differences in One Way ANOVAs of Psychological Distress and Positive Psychological Outcomes as a Function of Severity of Bushfire Exposure*

| Measure | Post-hoc group differences | *p* |
| --- | --- | --- |
| Anxiety | High > Medium | <.001 |
| (GAD7 Total)^1^ | High > Low | <.001 |
|  | High > Indirect | <.004 |
|  | High > Control | <.001 |
|  | Medium > Low | .051 |
|  | Medium = Indirect | .313 |
|  | Medium > Control | <.001 |
|  | Low < Indirect | <.001 |
|  | Low = Control | .976 |
|  | Indirect > Control | <.001 |
| Depression | High > Medium | <.001 |
| (PHQ9 Total) ^2^ | High > Low | <.001 |
|  | High > Indirect | <.001 |
|  | High > Control | <.001 |
|  | Medium > Low | .054 |
|  | Medium = Indirect | .429 |
|  | Medium > Control | .002 |
|  | Low < Indirect | <.001 |
|  | Low = Control | .982 |
|  | Indirect > Control | <.001 |
| Resilient Coping | High = Medium | .999 |
| (BRCS4 Total) ^2^ | High = Low | .497 |
|  | High = Indirect | .742 |
|  | High = Control | .978 |
|  | Medium = Low | .435 |
|  | Medium = Indirect | .433 |
|  | Medium = Control | .811 |
|  | Low > Indirect | .013 |
|  | Low > Control | .222 |
|  | Indirect = Control | .868 |
| Stress (PSS4 Total) ^2^ | High = Medium | .170 |
|  | High > Low | .003 |
|  | High = Indirect | .657 |
|  | High = Control | .063 |
|  | Medium = Low | .478 |
|  | Medium < Indirect | .005 |
|  | Medium = Control | .998 |
|  | Low < Indirect | <.001 |
|  | Low = Control | .607 |
|  | Indirect > Control | <.001 |
| Wellbeing (WHO5 %)^1^ | High > Medium | .033 |
|  | High > Low | <.001 |
|  | High > Indirect | <.001 |
|  | High > Control | <.001 |
|  | Medium = Low | .434 |
|  | Medium > Indirect | <.001 |
|  | Medium > Control | .011 |
|  | Low = Indirect | .160 |
|  | Low = Control | .800 |
|  | Indirect = Control | .544 |
| PTSD (PTSDI8) ^2^ | High > Medium | <.001 |
|  | High > Low | <.001 |
|  | Medium > Low | <.001 |

^1^ Tukeys

^2^ Games Howell

**Table 8.**

*Distress and Positive Psychological Outcomes for Bushfire-affected and Non-affected Participants.*

| Variable (survey measure) | Bushfire-affected | | | | Non-affected | | | |  | |  | |  |  |  |
| --- | --- | --- | --- | --- | --- | --- | --- | --- | --- | --- | --- | --- | --- | --- | --- |
|  | *N* *N* | *M* | *SD* | *N* | | *M* | *SD* | *df* | | *t* | | *p* | | Cohen’s *d* | Cronbach’s alpha |
| Depression (PHQ9) | 1593 | 9.6 | 7.0 | 1417 | | 8.5 | 6.9 | 3050 | | -4.27 | | **<.001** | | -.15 | .98 |
| Anxiety (GAD7) | 1594 | 8.2 | 5.7 | 1473 | | 7.3 | 5.6 | 3051 | | -2.64 | | **<.001** | | -.14 | .97 |
| Stress  (PSS4) | 1595 | 6.6 | 2.8 | 1476 | | 6.7 | 3.0 | 2988 | | 1.33 | | .184 | | .05 | .68 |
| Resilient-coping (BRCS4) | 1596 | 14.9 | 2.7 | 1478 | | 14.6 | 2.4 | 3068 | | -2.67 | | **.008** | | -.10 | .70 |
| Wellbeing (WHO5) | 1593 | 14.2 | 5.5 | 1477 | | 13.1 | 5.7 | 3068 | | -5.57 | | **<.001** | | -.20 | *.*89 |
|  |  |  |  |  | |  |  |  | |  | |  | |  |  |

## Table 9.

*Mean Psychological Distress and Positive Psychological Outcomes* *Scores by Gender*

| Variable (survey measure) | Gender | N | Mean | SD | SE | *p* | t | df | Cohen’s *d* |
| --- | --- | --- | --- | --- | --- | --- | --- | --- | --- |
| Resilience  (BRCS4) | Male | 1024 | 15.19 | 2.583 | .081 |  |  |  |  |
|  | Female, Other, PNS | 2054 | 14.54 | 2.575 | .057 | <.001 | 6.646 | 3076 | .25 |
| Stress  (PSS4) | Male | 1021 | 6.03 | 2.883 | .090 |  |  |  |  |
|  | Female, Other, PNS | 2052 | 6.97 | 2.882 | .064 | <.001 | -8.541 | 3071 | -.33 |
| Wellbeing  (WHO5) | Male | 1021 | 62.32 | 22.235 | .696 |  |  |  | .53 |
|  | Female, Other, PNS | 2050 | 50.94 | 21.389 | .472 | <.001 | 13.704 | 2431 |  |
| Anxiety  (GAD7) | Male | 1019 | 6.77 | 5.613 | .176 |  |  |  | -.26 |
|  | Female, Other, PNS | 2050 | 8.26 | 5.631 | .124 | <.001 | -6.900 | 3067 |  |
| Depression  (PHQ9) | Male | 1019 | 8.09 | 6.98 | .22 |  |  |  |  |
|  | Female, Other, PNS | 2046 | 9.59 | 6.88 | .15 | <.001 | -5.67 | 3063 | -.22 |
| PTSD  (PTSDI) | Male | 352 | 17.46 | 6.903 | .368 |  |  |  |  |
|  | Female, Other, PNS | 478 | 16.11 | 6.425 | .294 | .004 | 2.912 | 828 | .21 |

**Further details about cut-off lines in Figure 1.**

The cut-off lines in Figure 1 (main document) illustrate that regardless of bushfire exposure, respondents were generally distressed. Participants’ experiences of stress were higher than population means of 6 (Warttig et al., 2013). On average, participants demonstrated moderate resilient-coping, lower than overseas normative data (Kocalevent et al., 2017), and lower wellbeing than the average of 70% found in overseas normative data (Ellervik et al., 2014). Furthermore, a significant minority of participants had wellbeing scores below 50%, which is related to a higher risk of mortality (Topp et al., 2015). BRCS population data was obtained from a normative German sample (Kocalevent et al., 2017).

## Alternative psychological distress and positive psychological outcome ANOVA analyses with matched sample

We also conducted the descriptive analyses with a sample matched on age, gender, and SES, Findings were replicated, with the exception that resilient coping was no longer significantly different between bushfire-affected and non-affected groups.

**Table 10.**

*Matched Sample: Mean (SD) psychological Distress and Positive Psychological Outcome Scores as a Function of Severity of Bushfire Exposure*

|  | Control | Indirect | Low | Medium | | | High | |
| --- | --- | --- | --- | --- | --- | --- | --- | --- |
| GAD7 |  |  |  |  | | |  | |
| N | 819 | 394 | 400 | 520 | | | 296 | |
| Mean | 7.15 | 8.65 | 7.01 | 8.22 | | | 9.93 | |
| SD | 5.489 | 5.613 | 5.323 | 5.642 | | | 5.562 | |
| SE | .192 | .283 | .266 | .247 | | | .323 | |
| PHQ9 |  |  |  |  | | |  | |
| N | 819 | 393 | 400 | 519 | | | 296 | |
| Mean | 8.33 | 10.01 | 8.09 | 9.54 | | | 11.88 | |
| SD | 6.751 | 6.974 | 6.311 | 6.951 | | | 7.035 | |
| SE | .236 | .352 | .316 | .305 | | | .409 | |
| PSS4 |  |  |  |  | | |  | |
| N | 820 | 396 | 400 | 521 | | | 296 | |
| Mean | 6.70 | 7.26 | 6.32 | 6.81 | | | 7.07 | |
| SD | 3.067 | 2.812 | 2.961 | 2.755 | | | 2.484 | |
| SE | .107 | .141 | .148 | .12 | | | .14 | |
| BRCS |  |  |  |  | | |  | |
| N | 821 | 397 | 400 | 521 | | | 297 | |
| Mean | 14.66 | 14.55 | 14.95 | 14.64 | | | 14.42 | |
| SD | 2.314 | 2.7 | 2.48 | 2.70 | | | 3.26 | |
| SE | .08 | .12 | .12 | .19 | | | .05 | |
| WHO5 |  |  |  |  | | |  | |
| N | 821 | 396 | 400 | 520 | | | 296 | |
| Mean | 51.68 | 50.76 | 52.89 | 54.42 | | | 56.93 | |
| SD | 22.277 | 22.659 | 20.998 | 21.252 | | | 21.724 | |
| SE | .777 | 1.139 | 1.050 | .932 | | | 1.263 | |
| PTSDI8 |  |  |  |  | | |  | |
| N |  |  | 97 | 236 | | | 215 | |
| Mean |  |  | 11.71 | | 15.77 | 20.13 | |  |
| SD |  |  | 5.109 | 6.072 | | | 5.572 | |
| SE |  |  | .519 | .395 | | | .380 | |

## Table 11.

*Matched sample: Mean Psychological Distress and Positive Psychological Outcomes* *Scores by Gender*

|  | | | | | | |  | |  | |  | |  | |
| --- | --- | --- | --- | --- | --- | --- | --- | --- | --- | --- | --- | --- | --- | --- |
| Variable (survey measure) | Gender | N | Mean | SD | SE | p | | t | | df | | Cohen’s *d* | |  |
| Resilience  (BRCS4) | Male | 691 | 15.00 | 2.620 | .100 |  | |  | |  | |  | |  |
|  | Female, Other, PNS | 1745 | 14.52 | 2.600 | .062 | <.001 | | 4.120 | | 2434 | | .19 | |  |
| Stress  (PSS4) | Male | 688 | 6.15 | 2.896 | .110 |  | |  | |  | |  | |  |
|  | Female, Other, PNS | 1745 | 7.06 | 2.846 | .068 | <.001 | | -7.074 | | 2431 | | -.32 | |  |
| Wellbeing  (WHO5) | Male | 689 | 59.55 | 22.216 | .846 |  | |  | |  | | .43 | |  |
|  | Female, Other, PNS | 1744 | 50.35 | 21.249 | .509 | <.001 | | 9.494 | | 2431 | |  | |  |
| Anxiety  (GAD7) | Male | 686 | 6.52 | 5.349 | .204 |  | |  | |  | | -.36 | |  |
|  | Female, Other, PNS | 1743 | 8.49 | 5.606 | .134 | <.001 | | -7.894 | | 2427 | |  | |  |
| Depression  (PHQ9) | Male | 686 | 7.72 | 6.632 | .253 |  | |  | |  | |  | |  |
|  | Female, Other, PNS | 1741 | 9.86 | 6.908 | .166 | <.001 | | -6.957 | | 2425 | | -.32 | |  |
| PTSD  (PTSDI8) | Male | 211 | 16.40 | 6.626 | .456 |  | |  | |  | |  | |  |
|  | Female, Other, PNS | 397 | 16.17 | 6.471 | .325 | .34 | | .417 | | 606 | | -.04 | |  |

**3.3. Predictors of psychological distress and resilience among those affected by bushfires**

**Justification for factor analysis approach to combine outcome measures:**

Mental health outcomes after disaster can be both positive and negative. After a disaster, a significant minority of people can experience psychological distress (e.g, depression, anxiety, and PTSD), but the majority of people actually experience either a lack of psychological distress, or positive psychological outcomes (Bryant et al., 2021; Norris et al., 2002, Lowe et al., 2015, Li et al., 2012). These different mental health trajectories after disaster are consistent with research indicating that mental health is best represented as two correlated but distinct components: the absence of a mental disorder, and the presence of positive psychological wellbeing characteristics (Greenspoon & Saklofske, 2001; Keyes, 2005).

When measuring symptoms of psychological disorder after disaster, it is important to consider that there are high levels of comorbidity amongst disorders associated with depression, anxiety, and PTSD, and a person’s psychological symptoms can shift over time from one diagnosis to another (Meewisse et al, 2011). Research has also shown that there is a single underlying factor associated with disorders of depression, anxiety, and post-traumatic stress, characterised as internalising symptoms (Conway et al., 2021; Kotov et al., 2017). Measurement of positive psychological outcomes after disaster has only more recently become common, and little research been conducted regarding the shared meaning captured in scales measuring positive outcomes.

Aligning with the two-dimensional approach to mental health trajectories following disaster, we explored both positive and negative dimensions of mental health following bushfire. To understand the factors that predict resilience after disaster, given that a single psychological outcome measure does not capture a person’s overall distress or resilience and may miss distress that would be better captured by another scale, we elected to measure a range of common psychological distress and positive psychological experiences, to identify factors that are predictive of more general resilience, rather than single disorder.

**Preliminary Factor Analysis**

Preliminary analyses indicated high correlations among the dependent variables measuring depression (PHQ9), anxiety (GAD7), PTSD (PTSDI8), PSS4 (stress), resilient coping (BRCS), and wellbeing (WHO5). As such, we conducted a factor analysis to generate aggregate outcomes scores, distilling multiple symptom scores from common diagnostic scales known to have conceptually overlapping symptoms and constructs (e.g., depression, anxiety, PTSD, wellbeing), into overarching “psychological distress” and “positive psychological outcomes” constructs.

Prior to factor analysing scale scores, we conducted a preliminary Principal Components Analysis which indicated that the 37 items across 6 measures aligned sufficiently with factors representing each of the constructs that the scales were designed to measure. That is, we subjected the 37 item scores from our six outcome variables to an exploratory Principal Components Analysis with oblimin rotation (to allow for correlated factors) and Kaiser Normalization. Barlett’s test of sphericity suggested that the data were suitable for factor analysis, c^2^(666)=20437.15, *p*<.001. Communalities were sufficient for all 37 items (initial ≥1.00; extracted ≥.47).

The analysis yielded five factors with an eigenvalue of 1.0, explaining 65.2% of the variance. The first factor had an eigenvalue of 13.4 and explained 38.1% of the variance. Items that loaded on this factor included all of the PHQ9 and GAD7 items (> .68) and items 1 and 4 of the PSS4 (>.6). The second factor had an eigenvalue of 5.4 and explained 14.5% of the variance. Items that loaded on this factor included the 5 WHO5 items (> .58). The third factor had an eigenvalue of 2.8 and explained 7.6% of the variance. Items that loaded on this factor were the 8 PTSD items (> .76). The fourth factor had an eigenvalue of 1.6 and explained 4.2% of the variance. Items that loaded on this factor included all of the BRCS4 items (>.57). The fifth factor had an eigenvalue of 1.0 and explained 2.8% of the variance. Items that loaded on this factor (> .70) were Questions 2 and 3 of the PSS4 (reverse scored). PSS4 items 1 and 4 loaded with the PHQ9/GAD7 factor. The exploratory factor analysis demonstrated that items on each scale tended to represent separate factors, noting that PHQ9 and GAD7 items loaded together on one factor, and that two PSS4 items loaded with PHQ9 and GAD7 items (but note in the next section that the PSS4 is removed from subsequent analyses).

**Primary Factor Analysis**

To create final factor scores that could be explained in reference to scale origin (for reader clarity), we conducted a Principal Components Analysis of the scale scores, with oblique rotation (oblimin) and Kaiser Normalization. This yielded two factors with eigenvalues greater than 1, together explaining 73.2% of the variance. The PHQ-9, the GAD-7, and PTSDI-8 all loaded strongly on the first factor, which together represent the well-established shared concept of “Internalising Symptoms” (Kotov et al., 2017), and here we label “Psychological Distress [Distress]” (Kotov et al., 2017). The BRCS and WHO5 loaded strongly on the second factor, which we label “Positive Psychological Outcomes [Positive]”. The PSS4 had a cross-loading of .55 on Distress and -.55 on Positive and was subsequently removed from analyses. All other outcomes had cross-loadings below .2. The factor analysis confirmed a two-dimension approach to outcome measurement of mental health and wellbeing, and the factor scores for “Distress” and “Positive” outcomes were used for regression analyses. Scores on the Distress and Positive factors were significantly associated with one another (*r* = .113, *p* = .001).

**Table 12.**

*Factor Loadings for Factor Analysis of 6 Outcome Variable Total Scale Scores (BRCS4, PSS4, WHO5, GAD7, PHQ9, and PTSD).*

| \| *Pattern Matrix^a^* \| \| \| \| --- \| --- \| --- \| \|  \| Factor Loading \| \| \| Distress \| Positive \| \| BRCS4 \| .105 \| .775 \| \| PSS4 \| .550 \| -.554 \| \| WHO5 \| -.098 \| .841 \| \| GAD7 \| .891 \| -.158 \| \| PHQ9 \| .882 \| -.160 \| \| PTSDI8 \| .776 \| .341 \| \| Extraction Method: Principal Component Analysis.  Rotation Method: Oblimin with Kaiser Normalization. \| \| \| \| a. Rotation converged in 8 iterations. \| \| \| |
| --- | --- | --- | --- | --- | --- | --- | --- | --- | --- | --- | --- | --- | --- | --- | --- | --- | --- | --- | --- | --- | --- | --- | --- | --- | --- | --- | --- | --- | --- | --- | --- | --- |

**Data assumption testing for Regression analyses**

There was independence of residuals as assessed by a Durbin-Watson statistic of 1.83 (Psychological distress) and 1.94 (Positive psychological outcomes). There was linearity as assessed by visual inspections of plots between the studentized residuals against predicted values. All correlations between independent variables were below .7, and tolerance values were all greater than .1, therefore multicollinearity was not suspected. There were 4 Psychological distress and 4 Positive psychological outcomes outliers (standardized residuals above 3 standard deviations). Given the small number of outliers, and not having reason to question the validity of the scores, these cases were retained in the analysis.

In sum, assumptions were met for the analysis approach. Analyses were also run using a backward stepwise approach, with identical results.

**Table 13.**

*Pearson Correlations between Distress and Positive Psychological Outcomes and Demographic, Clinical, Financial, and Bushfire Experience Variables*

| Correlations | | | | | | | | | | | | | | | |
| --- | --- | --- | --- | --- | --- | --- | --- | --- | --- | --- | --- | --- | --- | --- | --- |
|  | | 1 | 2 | 3 | 4 | 5 | 6 | 7 | 8 | 9 | 10 | 11 | 12 | 13 | 14 |
| 1. Distress | *r* |  |  |  |  |  |  |  |  |  |  |  |  |  |  |
|  | *N* |  |  |  |  |  |  |  |  |  |  |  |  |  |  |
| 1. Positive | *r* | -.113* |  |  |  |  |  |  |  |  |  |  |  |  |  |
|  | *N* | 830 |  |  |  |  |  |  |  |  |  |  |  |  |  |
| 1. Age | *r* | -.435* | .101* |  |  |  |  |  |  |  |  |  |  |  |  |
|  | *N* | 830 | 830 |  |  |  |  |  |  |  |  |  |  |  |  |
| 1. Gender | *r* | .012 | -.287** | -.225** |  |  |  |  |  |  |  |  |  |  |  |
|  | *N* | 830 | 830 | 3082 |  |  |  |  |  |  |  |  |  |  |  |
| 1. Education | *r* | .018 | .247** | .164** | -.029 |  |  |  |  |  |  |  |  |  |  |
|  | *N* | 746 | 746 | 2500 | 2500 |  |  |  |  |  |  |  |  |  |  |
| 1. SES (IRSAD) | *r* | .025 | .139** | -.075** | -.074** | .067* |  |  |  |  |  |  |  |  |  |
|  | *N* | 776 | 776 | 2744 | 2744 | 2239 |  |  |  |  |  |  |  |  |  |
| 1. Remoteness | *r* | -.139** | -.182** | .162** | .035 | -.044* | -.428** |  |  |  |  |  |  |  |  |
|  | *N* | 776 | 776 | 2744 | 2744 | 2239 | 2744 |  |  |  |  |  |  |  |  |
| 1. Income | *r* | -.140** | .223** | -.018 | -.022 | .198** | .120** | -.090** |  |  |  |  |  |  |  |
|  | *N* | 746 | 746 | 2497 | 2497 | 2496 | 2236 | 2236 |  |  |  |  |  |  |  |
| 1. Income Loss | *r* | .009 | .019 | .009 | .035 | .088** | -.027 | .029 | .103** |  |  |  |  |  |  |
|  | *N* | 737 | 737 | 2465 | 2465 | 2464 | 2210 | 2210 | 2463 |  |  |  |  |  |  |
| 1. Financial Threat (FTS) | *r* | .455** | -.110* | -.188** | .042* | -.084** | -.027 | -.004 | -.196** | -.097** |  |  |  |  |  |
|  | *N* | 735 | 735 | 2474 | 2474 | 2474 | 2218 | 2218 | 2472 | 2442 |  |  |  |  |  |
| 1. Covid19 Stress (WSAS) | *r* | .336** | .098** | -.240** | -.033 | .028 | .081** | -.121** | -.047* | -.119** | .461** |  |  |  |  |
|  | *N* | 743 | 743 | 2494 | 2494 | 2492 | 2234 | 2234 | 2491 | 2460 | 2473 |  |  |  |  |
| 1. Pre-existing Physical Diagnosis | *r* | .240** | -.104** | -.022 | -.005 | -.012 | -.050* | .009 | -.125** | -.037 | .122** | .125** |  |  |  |
|  | *N* | 809 | 809 | 2850 | 2850 | 2493 | 2548 | 2548 | 2490 | 2458 | 2469 | 2488 |  |  |  |
| 1. Pre-existing Mental Health Diagnosis | *r* | .416** | -.204** | -.148** | .066** | -.042* | -.059** | -.002 | -.190** | -.044* | .289** | .186** | .434** |  |  |
|  | *N* | 793 | 793 | 2818 | 2818 | 2468 | 2520 | 2520 | 2464 | 2432 | 2445 | 2461 | 2818 |  |  |
| 1. Prior Fire Exposure | *r* | .147** | .145** | .111** | -.132** | .117** | -.049* | .144** | -.018 | -.041* | .033 | .125** | .137** | .109** |  |
|  | *N* | 824 | 824 | 3007 | 3007 | 2450 | 2694 | 2694 | 2446 | 2416 | 2426 | 2443 | 2793 | 2762 |  |
| 1. 2019/2020 Bushfire Exposure Scale | *r* | .396** | .260** | -.001 | -.186** | .072** | -.017 | .072** | -.033 | -.282** | .107** | .201** | .221** | .115** | .424** |
|  | *N* | 830 | 830 | 3083 | 3082 | 2500 | 2744 | 2744 | 2497 | 2465 | 2474 | 2494 | 2850 | 2818 | 3007 |

**. Correlation is significant at the 0.01 level (2-tailed

**Alternative regression analyses for individual scale outcomes**

We used a hierarchical multiple linear regression to determine if, after controlling for individual demographic variables (age, education, gender, income), COVID-19 stressors, and clinical and financial stressors (Step 1), severity of bushfire exposure (Step 2) contributed to the prediction of PHQ9 (Depression), GAD7 (Anxiety), PTSDI (PTSD), PSS4 (Stress), WHO5 (Wellbeing), and BRCS4 (Resilience) outcome scores. Significance of predictors in the final models are reported in Table 15. The unique differences across each of the specific scales compared to factor scores have implications for individual psychological treatment, which is beyond the scope of this paper.

| **Table 14.**  Significance of Variables as Predictors in 8 Separate Hierarchical Linear Regression Models Predicting Psychological Outcomes After Bushfire (6 Psychological Outcome Scales, and 2 Factor Scores) |
| --- |
|  |

|  | Scale Scores | | | | | | Factor Scores | |
| --- | --- | --- | --- | --- | --- | --- | --- | --- |
| Variable | PHQ9  (Depression) | GAD7  (Anxiety) | PTSDI  (PTSD) | PSS4  (Stress) | WHO5  (Wellbeing) | BRCS  (Resilience) | Distress Score | Positive  Score |
| Age | <.001 | <.001 | .025 | <.001 | .015 | <.001 | <.001 | .033 |
| Gender | .506 | .130 | .555 | <.001 | <.001 | <.001 | .625 | <.001 |
| Education | .029 | .703 | .062 | .023 | <.001 | .003 | .017 | <.001 |
| Income | .001 | .009 | .194 | .084 | .026 | .043 | .084 | .021 |
| Covid (WSAS) | .076 | .368 | .001 | .814 | <.001 | .771 | .530 | <.001 |
| Financial Threat (FTS) | <.001 | <.001 | <.001 | <.001 | <.001 | <.001 | <.001 | .005 |
| Pre-existing Mental Health Diagnosis | <.001 | <.001 | <.001 | <.001 | <.001 | <.001 | <.001 | .003 |
| Pre-existing Physical Health Diagnosis | .176 | .371 | .763 | .983 | .521 | .001 | .605 | .381 |
| Prior Fire Exposure | .085 | .011 | .038 | .995 | .428 | .045 | .859 | .730 |
| 2019/2020 Bushfire Exposure | .003 | .003 | <.001 | .008 | <.001 | <.001 | <.001 | <.001 |

|  |
| --- |

**References**

APA (2022) Psychologists struggle to meet demand amid mental health crisis. 2022 COVID-19 Practitioner Impact Survey. American Psychological Association. Available at: apa.org/pubs/reports/practitioner/2022-covid-psychologist-workload (accessed 12 December 22).

Australian Bureau of Statistics (2016) Socio-Economic Indexes for Areas. Available at: abs.gov.au/websitedbs/censushome.nsf/home/seifa (accessed 12 December 22).

Bountress KE, Gilmore AK, Metzger IW, et al. (2020) Impact of disaster exposure severity: Cascading effects across parental distress, adolescent PTSD symptoms, as well as parent-child conflict and communication. *Social Science & Medicine* 264: 113293.

Bryant RA, Gibbs L, Colin Gallagher H, et al. (2021) The dynamic course of psychological outcomes following the Victorian Black Saturday bushfires. *Australian & New Zealand Journal of Psychiatry* 55(7): 666-677.

Conway CC, Krueger RF, Cicero DC, et al. (2021) Rethinking the diagnosis of mental disorders: data-driven psychological dimensions, not categories, as a framework for mental-health research, treatment, and training. *Current Directions in Psychological Science* 30(2): 151-158.

David D, Mellman TA, Mendoza LM, et al. (1996) Psychiatric morbidity following Hurricane Andrew. *Journal of Traumatic Stress* 9(3): 607-612.

Elal G and Slade P (2005) Traumatic Exposure Severity Scale (TESS): a measure of exposure to major disasters. *Journal of Traumatic Stress* 18(3): 213-220.

Fox-Wasylyshyn SM and El-Masri MM (2005) Handling missing data in self-report measures. *Research in Nursing Health* 28(6): 488-495.

Gale T and Hawley C (2001) A model for handling missing items on two depression rating scales. *International Clinical Psychopharmacology* 16(4): 205-214.

Ginexi EM, Weihs K, Simmens SJ, et al. (2000) Natural Disaster and Depression: A Prospective Investigation of Reactions to the 1993 Midwest Floods. *American Journal of Community Psychology* 28(4): 495-518.

Greenspoon PJ and Saklofske DH (2001) Toward an integration of subjective well-being and psychopathology. *Social Indicators Research* 54(1): 81-108.

Ikizer G, Karanci A and Dogulu C (2016) How does impact of objective and subjective disaster exposure relate to the three clusters of posttraumatic stress symptoms? *Anatolian Journal of Psychiatry* 17(3).

Keyes CL (2005) Mental illness and/or mental health? Investigating axioms of the complete state model of health. *Journal of Consultulting and Clinical Psychology* 73(3): 539-548.

Kocalevent R-D, Zenger M, Hinz A, et al. (2017) Resilient coping in the general population: standardization of the brief resilient coping scale (BRCS). *Health and Quality of Life Outcomes* 15(1): 251.

Kotov R, Krueger RF, Watson D, et al. (2017) The Hierarchical Taxonomy of Psychopathology (HiTOP): A dimensional alternative to traditional nosologies. *Journal of Abnormal Psychology* 126(4): 454-477.

Li M, Xu J, He Y, et al. (2012) The analysis of the resilience of adults one year after the 2008 Wenchuan earthquake. *Journal of Community Psychology* 40(7): 860-870.

Lowe SR, Sampson L, Gruebner O, et al. (2015) Psychological resilience after Hurricane Sandy: The influence of individual- and community-level factors on mental health after a large-scale natural disaster. *PloS one* 10(5): e0125761.

McKnight PE (2007) *Missing Data A Gentle Introduction.* New York: Guilford Press.

Meewisse M-L, Olff M, Kleber R, et al. (2011) The course of mental health disorders after a disaster: Predictors and comorbidity. *Journal of Traumatic Stress* 24(4): 405-413.

Norris FH, Tracy M and Galea S (2009) Looking for resilience: Understanding the longitudinal trajectories of responses to stress. *Social Science & Medicine* 68(12): 2190-2198.

Norris FH and Uhl GA (1993) Chronic stress as a mediator of acute stress: The case of Hurricane Hugo. *Journal of Applied Social Psychology* 23(16): 1263-1284.

Polusny MA, Ries BJ, Schultz JR, et al. (2008) PTSD symptom clusters associated with physical health and health care utilization in rural primary care patients exposed to natural disaster. *Journal of Traumatic Stress* 21(1): 75-82.

Shrive FM, Stuart H, Quan H, et al. (2006) Dealing with missing data in a multi-question depression scale: A comparison of imputation methods. *BMC Medical Research Methodology* 6(1): 57.

Sumer N, Karanci AN, Berument SK, et al. (2005) Personal resources, coping self-efficacy, and quake exposure as predictors of psychological distress following the 1999 earthquake in Turkey. *Journal of Traumatic Stress* 18(4): 331-342.

Topp CW, Østergaard SD, Søndergaard S, et al. (2015) The WHO-5 Well-Being Index: A Systematic Review of the Literature. *Psychotherapy and Psychosomatics* 84(3): 167-176.

Verger P, Rotily M, Hunault C, et al. (2003) Assessment of exposure to a flood disaster in a mental-health study. *Journal of Exposure Science & Environmental Epidemiology* 13(6): 436-442.

Warttig SL, Forshaw MJ, South J, et al. (2013) New, normative, English-sample data for the Short Form Perceived Stress Scale (PSS-4). *Journal of Health Psychology* 18(12): 1617-1628.
